# *Let-7i* Reduces Aggressive Phenotype and Induces *BRCAness* in Ovarian Cancer Cells

**DOI:** 10.3390/cancers13184617

**Published:** 2021-09-15

**Authors:** Evgeny Chirshev, Tise Suzuki, Hanmin Wang, Anthony Nguyen, Nozomi Hojo, Linda Sanderman, Saied Mirshahidi, Yevgeniya J. Ioffe, Juli J. Unternaehrer

**Affiliations:** 1 Department of Basic Sciences, Division of Biochemistry, Loma Linda University, Loma Linda, CA 92354, USA; echirshev@students.llu.edu (E.C.); tsuzuki@students.llu.edu (T.S.); hwang1@students.llu.edu (H.W.); anthony_nguyen1994@yahoo.com (A.N.); nozomi.hojo@riken.jp (N.H.); Linda.Sanderman@csusb.edu (L.S.); 2 Department of Pathology and Human Anatomy, Loma Linda University School of Medicine, Loma Linda, CA 92354, USA; 3 Biology Department, California State University San Bernardino, San Bernardino, CA 92407, USA; 4 Biospecimen Laboratory, Loma Linda University Cancer Center, Department of Basic Sciences, Division of Microbiology & Molecular Genetics, Loma Linda University, Loma Linda, CA 92354, USA; smirshahidi@llu.edu; 5 Department of Gynecology and Obstetrics, Division of Gynecologic Oncology, Loma Linda University Medical Center, Loma Linda, CA 92354, USA; YIoffe@llu.edu; 6 Department of Gynecology and Obstetrics, Loma Linda University, Loma Linda, CA 92354, USA; 7 Center for Health Disparities and Molecular Medicine, Loma Linda University, Loma Linda, CA 92354, USA

**Keywords:** ovarian cancer, miRNA *let-7*, cancer stem cells, chemoresistance, *BRCA*ness

## Abstract

**Simple Summary:**

Ovarian cancer has a dismal prognosis and innovative treatment options are necessary to improve survival. Because the microRNA *let-7* is often lost in this and other cancers, and its loss is associated with poor prognosis, we focused on therapeutic strategies to replace it. We report that *let-7* overexpression in patient-derived cells resulted in a loss of aggressiveness: inhibition of migration and invasion (associated with metastasis), repression of cancer stem cell attributes (necessary for tumor maintenance and recurrence), and promotion of cell death (required for sensitivity to chemotherapy drugs). Further, cells in which *let-7* is overexpressed were more sensitive to PARP inhibitors, even in patients who otherwise could not benefit from these drugs. We show that *let-7* reduces the expression of several genes that may contribute to these effects. These actions of *let-7* add to the rationale for use of this miRNA as a treatment for selected ovarian cancer patients.

**Abstract:**

High-grade serous carcinoma of the ovary is a deadly gynecological cancer with poor long-term survival. Dysregulation of microRNAs has been shown to contribute to the formation of cancer stem cells (CSCs), an important part of oncogenesis and tumor progression. The *let-7* family of microRNAs has previously been shown to regulate stemness and has tumor suppressive actions in a variety of cancers, including ovarian. Here, we demonstrate tumor suppressor actions of *let-7i:* repression of cancer cell stemness, inhibition of migration and invasion, and promotion of apoptosis, features important for cancer progression, relapse, and metastasis. *Let-7i* over-expression results in increased sensitivity to the PARP inhibitor olaparib in samples without BRCA mutations, consistent with induction of *BRCA*ness phenotype. We also show that *let-7i* inhibits the expression of several factors involved in the homologous recombination repair (HRR) pathway, providing potential mechanisms by which the *BRCA*ness phenotype could be induced. These actions of *let-7i* add to the rationale for use of this miRNA as a treatment for ovarian cancer patients, including those without mutations in the HRR pathway.

## 1. Introduction

Ovarian cancer is the second most common gynecological malignancy in the USA, with 90% of cancers being epithelial ovarian cancers [1,2]. Most cases of epithelial ovarian cancer are of high-grade serous ovarian carcinoma (HGSOC) with a long-term survival rate of 30%. The majority (85%) of ovarian cancer patients respond well to initial therapy; however, about 75% relapse, and this results in poor prognosis and survival [3,4]. At least in part, tumor recurrence/progression after initial therapy is due to the existence of a population of cancer stem-like cells within the tumor present at the inception of treatment. Cancer stem cells (CSCs) are also partially responsible for the maintenance and growth of tumors [5].

The first-line treatment of ovarian cancer involves primary surgical debulking treatment followed by six to eight cycles of combination chemotherapy, typically of platinum-based and taxane agents, sometimes with the addition of VEGF inhibitor bevacizumab [6]. If upfront debulking surgery is not undertaken, the protocol consists of neoadjuvant combination of typically platinum/taxane agents, followed by interval debulking surgery and additional chemotherapy administration. While initially effective, the majority of patients develop recurrence at the rate of 60–85% [7]. Grossly visible residual disease following debulking surgery is a negative predictor for survival, among other factors, such as patient’s age, tumor grade, and pre-surgical tumor burden [3,8,9].

Because chemotherapy drugs cause DNA damage, it is therapeutically desirable to thwart DNA repair mechanisms. The homologous recombination repair (HRR) mechanism of DNA utilizes the undamaged copy of the gene as a template to repair the damaged DNA copy [10]. Poly-ADP ribose polymerase inhibitors (PARPi) have been demonstrated to be an effective targeted therapy, particularly in patients with homologous recombination deficiency (HRD), acquired either as a germline mutation carrier status or as somatic mutations, i.e., in the tumor itself. One of the causes of HRD is inactivation mutations in *BRCA*1/2 genes, either germline or somatic. PARPi suppress the alternative nuclear DNA repair pathway. HRD cells are unable to repair using homologous recombination (HR), and cell lethality is induced [11]. Apart from mutations in *BRCA*1/2 genes, other defects in HRR induce sensitivity to PARPi. This phenotype is termed as *BRCA*ness [10]. While the patients with an intact HRR pathway are candidates for PARPis, their progression-free survival and response rates are less favorable than those seen in patients with HRD, when treated in the same clinical circumstances [12,13,14,15]. In patients with *BRCA* mutations treated with the PARPi olaparib in maintenance setting for platinum-sensitive ovarian cancer after at least two lines of platinum-based chemotherapy, SOLO2 trials demonstrated an advantage in overall survival of 13 months [16]. Inducing *BRCA*ness phenotype in patients without *BRCA*1/2 mutations can be an effective solution to improve progression-free survival, response to targeted therapy, and possibly even overall survival.

In the modern era, molecular profiling of ovarian cancers has become widely utilized both in the upfront and recurrent treatment settings, with the goal of utilization of targeted therapy. PARPi have become the quintessential compound illustrating the utility of germline and somatic tumor testing. These agents are utilized both as maintenance treatment, sometimes in combination with bevacizumab, and as monotherapy in heavily pretreated recurrent disease [17,18]. PARPi have also been widely studied in clinical trials in combination with VEGF inhibitors, immunotherapy, and other targeted agents, in particular cediranib, a VEGF receptor and PDGF tyrosine kinase inhibitor [19].

MicroRNAs (miRNA) are small, ~22 nucleotide, non-coding RNAs that regulate gene expression post-transcriptionally by binding the 3′ UTR of mRNA targets [20]. MiRNAs are dysregulated in HGSOC, which affects response to chemotherapy-induced apoptosis [21]. *Let-7* (*lethal-7*) miRNA was first discovered in *C*. *elegans* as a heterochronic RNA regulating the transition from L4 into the adult worm [22]. *Let-7* regulates stem cell differentiation, as demonstrated by the failure of mutant worms to transition into adults, while over-expression causes premature development [23]. *Let-7* is essential to maintain somatic cells in the differentiated state. This miRNA must be repressed for somatic cell reprogramming to pluripotency [24,25,26]. It is repressed in many types of cancer, including ovarian, associated with an increase in stemness and poor prognosis [27,28,29]. While the regulation of *let-7* is incompletely understood, transcriptional, post-transcriptional, and epigenetic regulation are known to occur [30]. Factors that decrease levels of *let-7* have also been shown to increase the stemness, invasiveness, and chemoresistance of cancer cells [31,32,33]. In our previous work, we demonstrated that patient-derived (PD) samples with low levels of *let-7* correlate with increased self-renewal, pluripotency, and tumor burden [34]. Thus, therapeutically replacing *let-7* could be a strategy to combat cancer cell aggressiveness.

Since *let-7* is a known tumor suppressor microRNA, its up-regulation results in repression of a variety of its target oncogenes. Among them, *LIN28A* and *HMGA2* have been implicated in cancer initiation, progression, and chemoresistance, and both serve as markers of CSCs [35,36]. *LIN28A* is a pluripotency factor expressed in embryonic stem cells (ESCs); its levels decrease during differentiation [37]. Carcinomas that highly express *LIN28A* are associated with poor survival, and *LIN28A* inhibition by *let-7* results in reduced in vivo tumor progression [38,39]. *HMGA2* is a chromatin-associated protein that facilitates gene transcription by binding A/T rich sequences in proximity to the binding sites of specific transcription factors and modifying chromatin structure. *HMGA2* facilitates embryonic stem cell exit from naïve to ground state, and is highly expressed during embryonic development, but is absent in differentiated cells [37,40]. *HMGA2* is essential for ESC exit from pluripotency and for the onset of differentiation [40].

There are 13 *let-7* family members encoding nine mature miRNAs. All have the same seed sequence and are thought to function in a similar manner. Evidence suggests that the slight differences in *let-7* sequences change target preference, leading to slightly different functions [41]. While some *let-7* family members act as tumor suppressors, others act as oncogenes [42,43,44]. *Let-7* was also shown to be involved in double strand DNA break repair by directly or indirectly repressing several involved factors including *BRCA1, RAD51, PARP, E2F1,* and *IGF1* [27,41,42,45,46,47]. Here, we focus on *let-7i,* one member of the *let-7* family, due to its repression to a greater extent than other *let-7* family members in our samples [34]. We demonstrate that *let-7i* up-regulation results in decreased stemness and self-renewal, reduced anchorage-independent growth, decreased functional phenotypes associated with cancer metastasis, increased apoptosis, and increased chemosensitivity to olaparib in *BRCA* wild-type (WT) samples.

## 2. Results

### 2.1. Let-7 Over-Expression in PD HGSOC Samples

**Figure 1:**
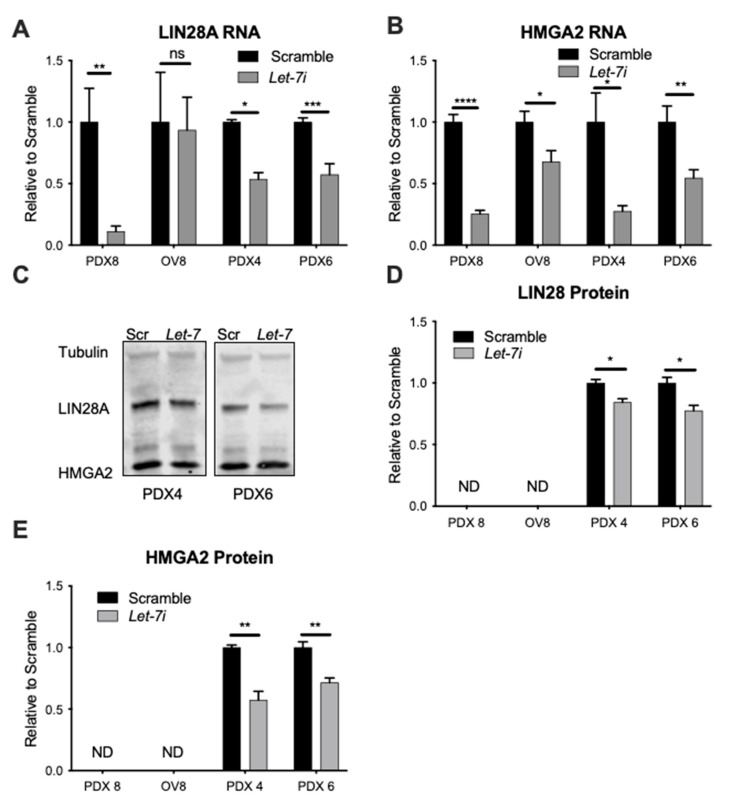
*Let-7i* up-regulation results in target repression. *Let-7i* mimic transfection resulted in repression of its targets *LIN28A* and/or *HMGA2* on RNA (**A**,**B**) and protein (**C**) level, quantified in (**D**,**E**). LIN28A and HMGA2 protein levels were not detected (ND) in PDX8 and OVCAR8 samples due to very low expression. Student’s *t* test was used for statistical analysis. N = 3+ independent replicates. Error bars: standard error of the means (SEM). * *p* ≤ 0.05, ** *p* ≤ 0.01, *** *p* ≤ 0.001, **** *p* ≤ 0.0001. *p* Value ≤ 0.05 was considered significant. ns, not significant.

Since both *LIN28A* and *HGMA2* are established targets of *let-7*, a decrease in their levels provides confirmation of *let-7i* over-expression. Upon transient *let-7i* over-expression in PD samples and OVCAR8 cell line (Supplementary Figure S1), we observed repression of these targets on RNA (Figure 1A,B) and protein (Figure 1C–E) level. Full blot is shown in Supplementary Figure S2. *Let-7i* is lost to varying extents in ovarian cancer cells: levels are highest in OVCAR8, lower in PDX8, then PDX6, and lowest in PDX4 [34]. Because *let-7* levels are comparatively higher, and pluripotency markers lower, in OVCAR8 [34], reductions in *LIN28A* and *HMGA2* were modest on RNA level, and protein was not detected, in this cell line. *Let-7* suppression of both *LIN28A* and *HMGA2* in PD HGSOC samples is consistent with reduction of stemness.

### 2.2. Let-7 Represses Self-Renewal

**Figure 2:**
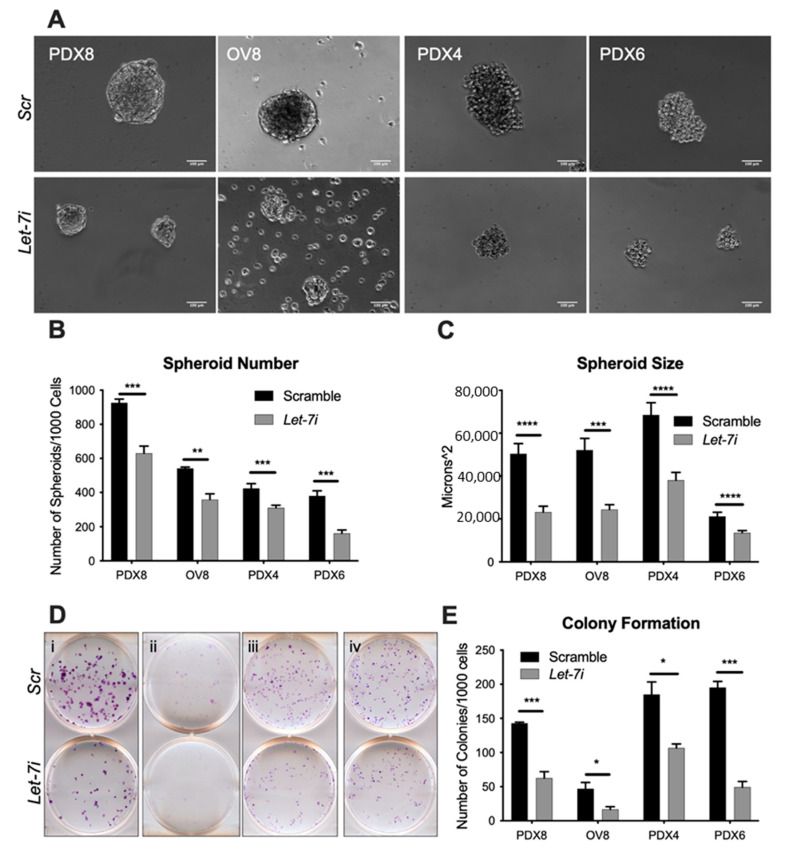
*Let-7* represses self-renewal. Cells were grown in spheroid conditions. (**A**) Images of spheroids from cells as indicated with control (Scr, upper panel) or *let-7i* overexpression (lower panel). Scale bar: 100 µm. *Let-7* transfection reduces number (**B**) and size (**C**) of spheroids. *Let-7* over-expression results in reduced ability to form colonies (**D**). (**E**) i, ii, iii, and iv represent PDX8, OV8, PDX4, and PDX6, respectively. N = 3+ independent replicates. OV8, OVCAR8. Student’s *t* test was used for statistical analysis. Error bars: SEM. * *p* ≤ 0.05, ** *p* ≤ 0.01, *** *p* ≤ 0.001, **** *p* ≤ 0.0001. *p* value ≤ 0.05 was considered significant.

While reduction of pluripotency factors suggests decreased stemness (Figure 1), the degree of reduction in functional terms is a more definitive measure. The self-renewal ability of cancer cells reflects their level of stemness and can be measured by the ability to form spheroids and colonies. A higher number of spheroids indicates a greater number of cancer stem cells present [48,49,50]. *Let-7* up-regulation resulted in reduced number and size of spheroids (Figure 2A–C) and reduced number of colonies formed (Figure 2D,E). PDX8, OVCAR8, PDX4, and PDX6 are represented by i, ii, iii, and iv, respectively. Together with reduced *LIN28A* and *HMGA2* expression, the reduced ability to form spheroids and colonies demonstrates the negative effect *let-7* has on the number of cancer stem cells present within these populations.

### 2.3. Let-7 Represses Migration and Invasion of HGSOC

**Figure 3:**
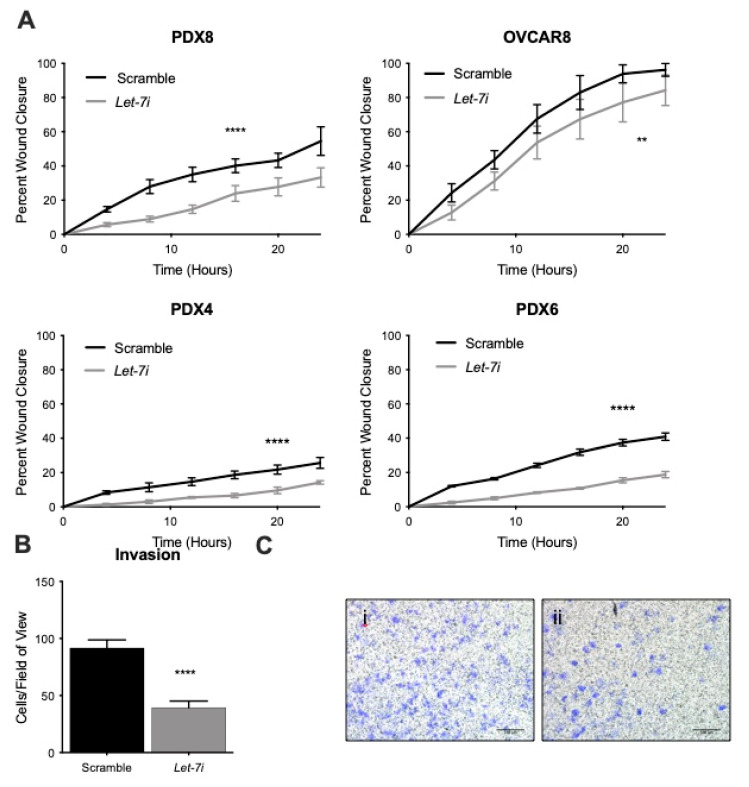
*Let-7* reduces migration and invasion. *Let-7* up-regulation demonstrates reduced ability to migrate (**A**) and invade (**B**,**C**). N = 3+ independent replicates. (**C**) i and ii represent Scramble and *Let-7i*, respectively. N = 3 independent replicates. Scale bar: 100 µm. Error bars: SEM. ** *p* ≤ 0.01, **** *p* ≤ 0.0001. *p* value ≤ 0.05 was considered significant.

To deduce the effect *let-7* has on the functional phenotype of ovarian cancer cells, we utilized migration and invasion assays. Migration is an important factor in cancer metastasis. We analyzed the cell migration for 24 h upon *let-7i* over-expression and demonstrated a reduced kinetics of cell migration (Figure 3A and Supplementary Figure S3). While migratory abilities are important for cancer metastasis, invasion through the basement membrane is required for widespread dissemination [51]. Matrigel invasion assays were used to assess ability to invade. Significant reduction in invasion was seen in OVCAR8 with *let-7* over-expression (Figure 3C and Supplementary Figure S4). Thus, *let-7i* overexpression led to reduced migratory and invasive abilities.

### 2.4. Let-7i Enhances HGSOC Sensitivity to PARP Inhibitors

**Figure 4:**
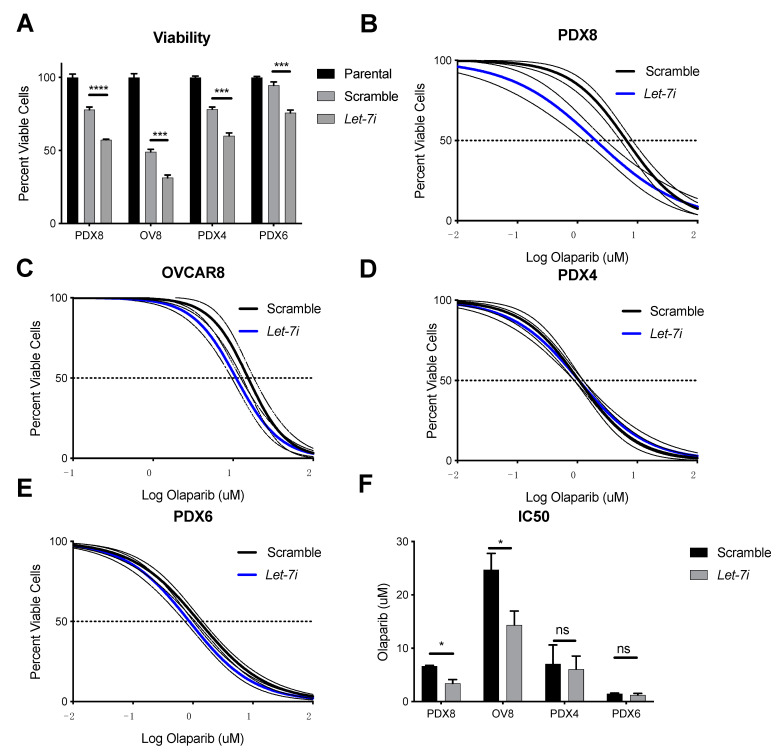
*Let-7i* effect on cancer cell viability and sensitivity to olaparib. *Let-7i* up-regulation resulted in reduced cell viability (**A**) and sensitivity to olaparib in HR competent samples (**B**,**C**). There was no effect on olaparib resistance in samples with BRCA1/2 mutation (**D**,**E**). (**F**) demonstrates IC50. N = 3+ independent replicates. Student’s *t* test was used for statistical analysis. Error bars: SEM. * *p* ≤ 0.05, *** *p* ≤ 0.001, **** *p* ≤ 0.0001. *p* value ≤ 0.05 was considered significant. ns, not significant.

CSCs have been implicated in increased resistance to standard chemotherapies [52]. Because we observed that *let-7i* reduced pluripotency and self-renewal ability (Figure 1 and Figure 2), we hypothesized that *let-7i* would also affect cell death, and found that increased *let-7i* expression alone was sufficient to significantly reduce cancer cell viability (Figure 4A). This effect was due to the induction of apoptosis (Supplementary Figure S5). Since PARP inhibitors are used as an adjuvant therapy to increase cancer cell death, we tested *let-7i* over-expression coupled with olaparib, an inhibitor of PARP. Two of our patient-derived samples, PDX4 and PDX6, possess mutations in the *BRCA* gene, which confers deficiency in homologous recombination repair (HRR). Two other samples, OVCAR8 and PDX8, demonstrated competent HRR [34]. *Let-7i* over-expression in PDX8 (Figure 4B) and OVCAR8 (Figure 4C) resulted in increased sensitivity to olaparib, while increased expression showed no effect in PDX4 (Figure 4D) and PDX6 (Figure 4E). Figure 4F represents reduction of chemoresistance represented by IC50, the drug concentration needed to decrease cell viability by fifty percent, by *let-7i* overexpression.

### 2.5. Let-7i Represses Factors Involved in HR Repair

**Figure 5:**
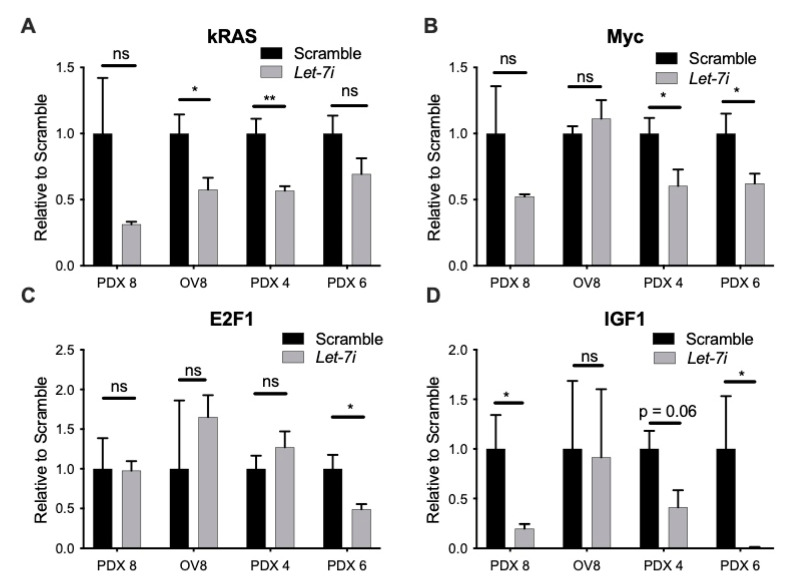
*Let-7* represses targets involved in HR pathway. RT-qPCR demonstrates *let-7i* effect on *KRAS* (**A**), *MYC* (**B**), *E2F1* (**C**), and *IGF1* (**D**). Student’s *t* test used for statistical analysis. N = 3+ independent replicates. Error bars: SEM. * *p* ≤ 0.05, ** *p* ≤ 0.01. *p* value ≤ 0.05 was considered significant. ns, not significant.

To determine the mechanism by which *let-7i* induces sensitivity to PARPi, we tested RNA levels of several known *let-7* targets that play an important role in the HR repair pathway. Some of the targets include *KRAS*, *MYC*, *IGF1*, and *E2F1* [53,54,55,56] As expected, *let-7i* over-expression resulted in repression of one or more of these targets on RNA level (Figure 5A–D), and protein level of cMYC (Supplementary Figure S6), demonstrating its effect via several different pathways.

## 3. Discussion

In this study, we demonstrated *let-7i*’s effect on pluripotency factor and CSC markers *HGMA2* and *LIN28A* (Figure 1), revealing its action to reduce stemness of HGSOC. It has been shown that *let-7* acts as a tumor suppressor by targeting pluripotency factors, including *HMGA2* [57,58]. We utilized an experimental system that is more clinically relevant than cell lines: low passage patient-derived samples. By targeting proteins involved in pluripotency, *let-7i* effectively decreases the population of CSCs within the samples, demonstrated by the decreased ability to form colonies and spheroids (Figure 2). CSCs have been implicated in cancer progression, relapse, and chemoresistance [5,59].

Stemness in cancer cells is complex and multifactorial. Although some of the cells we described here (PDX8 and OVCAR8) form a high number of spheres, indicating their self-renewal ability, these same cells do not express high levels of stem cell markers LIN28A and HMGA2. Thus, other factors must explain the stemness attributes in these cells. Other candidates affecting self-renewal include MYC, EGFR, RAS, and EZH2 [60,61,62,63].

Distant metastasis remains the major cause for cancer-associated mortalities [64]. In order for this to occur, cancer cells must invade through the basement membrane and migrate through the tissues [65]. Here we show that *let-7i* represses the ability of ovarian cancer cells to migrate and invade (Figure 3). *Let-7* targets (and downregulates) *HMGA2, STAT3, PKM2, PBX3, KRAS, E2F1, ITGB3,* and *MAP4K3*, which may explain its ability to decrease these properties [41,46,66,67,68,69,70,71].

In recent years, PARPis have emerged as a class of potent treatment compounds most effective in tumor cells deficient in HR, one type of DNA damage repair. *Let-7* targets several components of the HRR pathway, loss of which may therefore be an important factor in response to therapies targeting DNA damage repair, and *let-7* levels may predict response to PARPi. PARP-1, the founding and most abundant member of a family of highly conserved enzymes, along with PARP-2, have an important role in signaling DNA single strand breaks (SSB). Inhibition of PARP activity thus leads to an accumulation of SSBs that convert to double strand breaks (DSB), leading to cell death unless repaired by HR. *Let-7i* overexpression results in increased sensitivity to olaparib in BRCA WT samples (Figure 4). This effect is possibly via repression of HR-associated components including *KRAS, MYC, E2F1,* and *IGF,* shown to be repressed with *let-7* overexpression on the RNA level (Figure 5) and on the protein level in the case of c-MYC in two of four cell types (Supplementary Figure S6) [53,54,55,56]. Future studies will explore the mechanism of *let-7* effect on *BRCA*ness in more depth. PARPi therapy is most effective for patients with mutations in genes involved in HRR. By targeting and repressing several pathways involved in HRR, *let-7* becomes a potential treatment that can be used to induce *BRCA*ness phenotype in order to increase PARPi treatment efficacy in individuals without mutations in HRR genes.

Research on the molecular characterization of ovarian cancer on genomic, proteomic, and other levels has been ongoing for over a decade, launched by the flagship The Cancer Genome Atlas (TCGA) project [72]. Various scientific efforts have resulted in characterization of chromosomal aberrations, genomic rearrangements, and signaling pathway disruptions, as well as post-translational modifications [73,74]. Clinically, this has resulted in the development of tumor agnostic clinical trials, i.e., KEYNOTE trials [75,76]. Characterization of individual HGSOC tumors with these technologies allows for selection of individual therapy. In this investigation, we focused on the influence of miRNA *let-7i* on the HRR pathway and showed that *let-7i* overexpression contributes to the *BRCA*ness phenotype, and ultimately targeted therapy sensitivity.

In this study, we have demonstrated the effect micro-RNA *let-7i* has on ovarian cancer phenotype and treatment. Ovarian cancer is a very complex disease that involves abnormalities in different aspects and levels of cellular functions. Because of this, a multidisciplinary approach must be utilized in order to diagnose and treat the disease more successfully. Combination of genomics, epigenomics, transcriptomics, and proteomics will provide us with a more complete picture of the disease progress and the treatment options most likely to succeed. Experts from multiple disciplines of medicine and research working together to treat patients will facilitate progress and enhance patient outcomes [17,73,77,78].

## 4. Materials and Methods

### 4.1. Cell Culture

Tumor tissues were derived as described [34]; briefly, after informed consent, tumors were collected by Loma Linda University Cancer Center Biospecimen Laboratory. Of the set of eight patient-derived samples with in vitro growth characteristics that were conducive to these studies, the three with lowest *let-7* levels were selected for these *let-7i* overexpression experiments. Cells from patient tumors were cultured in 75% Ham’s F12 (all media from Fisher Scientific, Waltham, MA, USA), 25% DMEM with 5% fetal bovine serum (FBS; Omega Scientific, Tarzana, CA, USA), 10 μM insulin (chemicals are from Millipore/Sigma unless otherwise stated), 0.4 μM hydrocortisone, 2 μg/mL isoprenaline, 24 μg/mL adenine, and 100 U/mL penicillin, 10 μg/mL streptomycin (pen/strep). Then, 5–10 μM Y27632 (Peprotech, East Windsor, NJ, USA) was added to initial cultures [79]. Patient-derived cells were used at passage 15 or less.

Cell lines: OVCAR8 (human ovarian cancer; gift from Carlotta Glackin) were cultured in DMEM, 10% FBS, 2 mM L-glutamine, 0.1 mM BME, and pen/strep; and NCCIT (embryonal carcinoma, from George Daley) in RPMI, 10% FBS, 2 mM L-glutamine, 1% non-essential amino acids, 1 mM sodium pyruvate, and pen/strep.

### 4.2. MicroRNA Let-7 Overexpression

MiR-*let-7i* over-expression was achieved via lipofectamine (cat. 13778030, Life Technologies) transfection of mimics and scramble control purchased from IDTDNA. Up-regulation was confirmed via RT-qPCR.

- Scramble S: 5′-mCmArUmArUmUrGmCrGmCrGmUrAmUrAmGrUmCrGC.
- Scramble AS: 5′-/5Phos/rGrCrGrArCrUrArUrArCrGrCrGrCrArArUrArUmGmGrU-3′.
- Let-7i-5P S: 5′-mCmArGmCrAmCrAmArAmCrUmArCmUrAmCrCmUrCA-3′.
- Let-7i-5P AS: 5′-/5Phos/rUrGrArGrGrUrArGrUrArGrUrUrUrGrUrGrCrUmGmUrU-3′.

### 4.3. Real-Time Quantitative Reverse-Transcription PCR (RT-qPCR)

Total RNA from cell culture samples was isolated using TRIzol reagent (cat. 15596018, Life Technologies, Carlsbad, CA, USA) according to the manufacturer’s instructions. For mRNA expression analysis, cDNA was synthesized with 1 μg of total RNA using Thermo Scientific^TM^ Maxima First Strand cDNA Synthesis Kit for RT-qPCR, with dsDNAse (K1672; ThermoFisher Scientific, Waltham, MA, USA). Real-time RTq-PCR for mRNA was performed using Applied Biosystems^TM^ PowerUP^TM^ SYBR^TM^ Green Master mix (A25778; Thermo Fisher Scientific, Waltham, MA, USA) and specific primers on a Stratagene Mx3005P qPCR System (Model: 401513; Agilent Technologies, Santa Clara, CA, USA). Primer sequences are shown in Supplementary Table S1. For analysis of miRNA expression, cDNA was synthesized using 100 ng of total RNA with TaqMan primers (Life Technologies 4440887; let-7i assay 002221, U47 assay 001223) and Applied Biosystems^TM^ TaqMan^TM^ microRNA Reverse Transcription Kit (4366596; Thermo Fisher Scientific, Waltham, MA, USA). For real-time RT-qPCR for miRNA, Applied Biosystems^TM^ TaqMan^TM^ UniversalMaster Mix II (4440048; Thermo Fisher Scientific, Waltham, MA, USA) with TaqMan^TM^ probes (Life Technologies) were used. Results were analyzed using the ΔΔ cycles to threshold (ΔΔcT) method.

### 4.4. Spheroid Formation Assay

Spheroid formation assays were done as described [34]. Briefly, cells were plated at 1000 cells/mL in non-tissue culture coated plates (Olympus) and maintained for seven days in spheroid media (DMEM/F12 50/50, 0.4% bovine serum albumin, 10 ng/mL FGF, 20 ng/mL EGF, 6.7 ng/mL selenium, 5.5 μg/mL transferrin, 10 μg/mL insulin, and 1% knock out serum replacement (Gibco/ThermoFisher Scientific, Waltham, MA, USA)). Number and size of spheroids was then analyzed from phase contrast images using ImageJ software (National Institutes of Health, Bethesda, MD, USA).

### 4.5. Scratch Assay (Wound Healing Cell Migration Assay)

Wound healing assays were done as described [34]. Briefly, confluent cultures in 24-well cell culture plates were treated with mitomycin C (cat. S8146, Selleck Chemicals), scratchers were made with a 10 μL pipet tip, and phase contrast images of several positions along the length of the scratch were taken every four hours for 24 h with a Nikon Eclipse Ti microscope using MicroManager [80], analyzed with ImageJ (National Institutes of Health, Bethesda, MD, USA).

### 4.6. Western Blot

Lysates of cells in Laemmli buffer were sonicated, proteins were separated by SDS-PAGE and transferred to a 0.45 µM PVDF membrane (Fisher Scientific, Waltham, MA, USA). Membrane was blocked with 0.1–5% milk in TBST for 1 h. Primary antibodies were applied at 1:1000 dilution overnight, followed by immunoblotting. The membrane was stained with the antibodies at the same time given that the diluents were compatible and the location of the bands was detected individually in a prior experiment. Secondary antibodies (anti-rabbit IgG conjugated to Dylight 680, Fisher Scientific, Waltham, MA, USA) were applied for an hour at 1:30,000 dilution. Primary antibodies (Cell Signaling Technology, Danvers, MA, USA) included α/β-tubulin (21485), LIN28A (39785), and HMGA2 (81795). Secondary antibody immunoblotting was done with anti-rabbit IgG conjugated with Dylight 680 or 800 (Invitrogen, Carlsbad, CA, USA). Membranes were imaged with LI-COR Odyssey CLx Infrared Imaging System (LI-COR Biosciences, Lincoln, NE, USA) and analyzed with ImageJ software (National Institutes of Health, Bethesda, MD, USA). Full Western blots are shown in Supplementary Figure S2.

### 4.7. Invasion Assay

Invasion assay was derived based on a previously published protocol [81], as described [34]. Briefly, cells were cultured for 24 h in their respective media without FBS, then dissociated as usual with 0.05% trypsin/EDTA, resuspended in serum-free media, and 20,000 cells (PDX4 and PDX6) or 50,000 cells (PDX8 and OVCAR8) were plated on inserts. Cell number was optimized for proliferation rate; for quantification, samples with 50,000 cells were normalized to 20,000. Transwell inserts (Genesee Scientific, San Diego, CA, USA) were coated with 0.1 μg Basement Membrane Extract (BME) (3433-010-01) in 1X Cultrex coating buffer (3455-096-03) (Trevigen, Gaithersburg, MD, USA). Complete media (containing FBS) was placed in a lower chamber. After 24 h, the tops of inserts were wiped clean with a cotton tipped applicator, inserts were fixed in 70% ethanol for 15 min, stained in 0.2% crystal violet for 10–15 min, rinsed with distilled H_2_O, and allowed to dry. Cells were imaged (100×) using Leica DMi1 inverted microscope and counted using ImageJ.

### 4.8. Statistical Analysis

GraphPadPrism version 7.0 (GraphPad Software, La Jolla, CA, USA) was used to prepare figures and for statistical analysis. Statistical analysis methods are described in figure legends.

## 5. Conclusions

We have demonstrated repressive actions of *let-7i* on HGSOC cell stemness, resulting in reduced ability to self-renew, a feature necessary for cancer recurrence. We have also demonstrated *let-7i* actions on cancer metastasis by repressing the ability to migrate and invade. *Let-7i* actions on chemoresistance to PARPi olaparib is more complex. While *let-7i* has no effect on chemoresistance to olaparib in samples with *BRCA1/2* mutation, its upregulation in wild type samples resulted in increased sensitivity. The induction of sensitivity is presumed to be due to the repression of one or more of the factors involved in the HRR pathway including *KRAS*, *MYC, E2F1,* and *IGF1*. Taken together, these data suggest the possibility of using *let-7i* as an adjunct to standard therapy as well as an addition to PARPi for patients that do not have *BRCA1/2* mutations.

## Data Availability

Data will be made available from the corresponding author upon reasonable request.

## Supplementary Materials

The following are available online at https://www.mdpi.com/article/10.3390/cancers13184617/s1, Figure S1: *Let-7* transfection, Figure S2: Full images of Western blots, Figure S3: Images from wound healing assays, Figure S4: *Let-7* reduces invasion, Figure S5: *Let-7* increases apoptosis, Figure S6: *Let-7* effect on cMYC protein level, Table S1, RT-qPCR Primers.
