# Supplementary material for: Let-7i Reduces Aggressive Phenotype and Induces BRCAness in Ovarian Cancer Cells
Source: Cancers (Basel). 2021 Sep 15;13(18):4617. doi: 10.3390/cancers13184617 (PMC8468164; doi:10.3390/cancers13184617)
Supplement: Supplementary file 1 [file cancers-13-04617-s001.zip › cancers-1305628-supplementary.pdf]

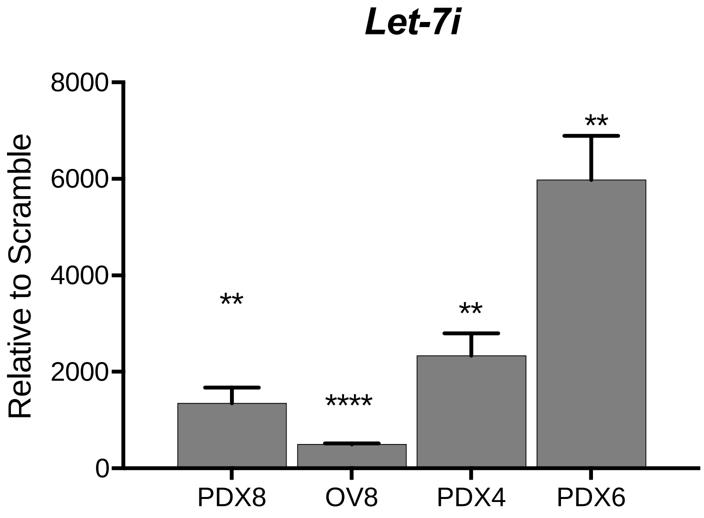

Figure S1. Let-7i transfection Lipofectamine transfection of let-7i mimic resulted in up-regulation of mature let-7i relative to scramble control. Let-7i levels in parental cells relative to each other have been previously described 1. Error bars: SEM. $^{**}p \leq 0.01$, $^{****}p \leq 0.0001$. p Value ≤ 0.05 was considered significant.

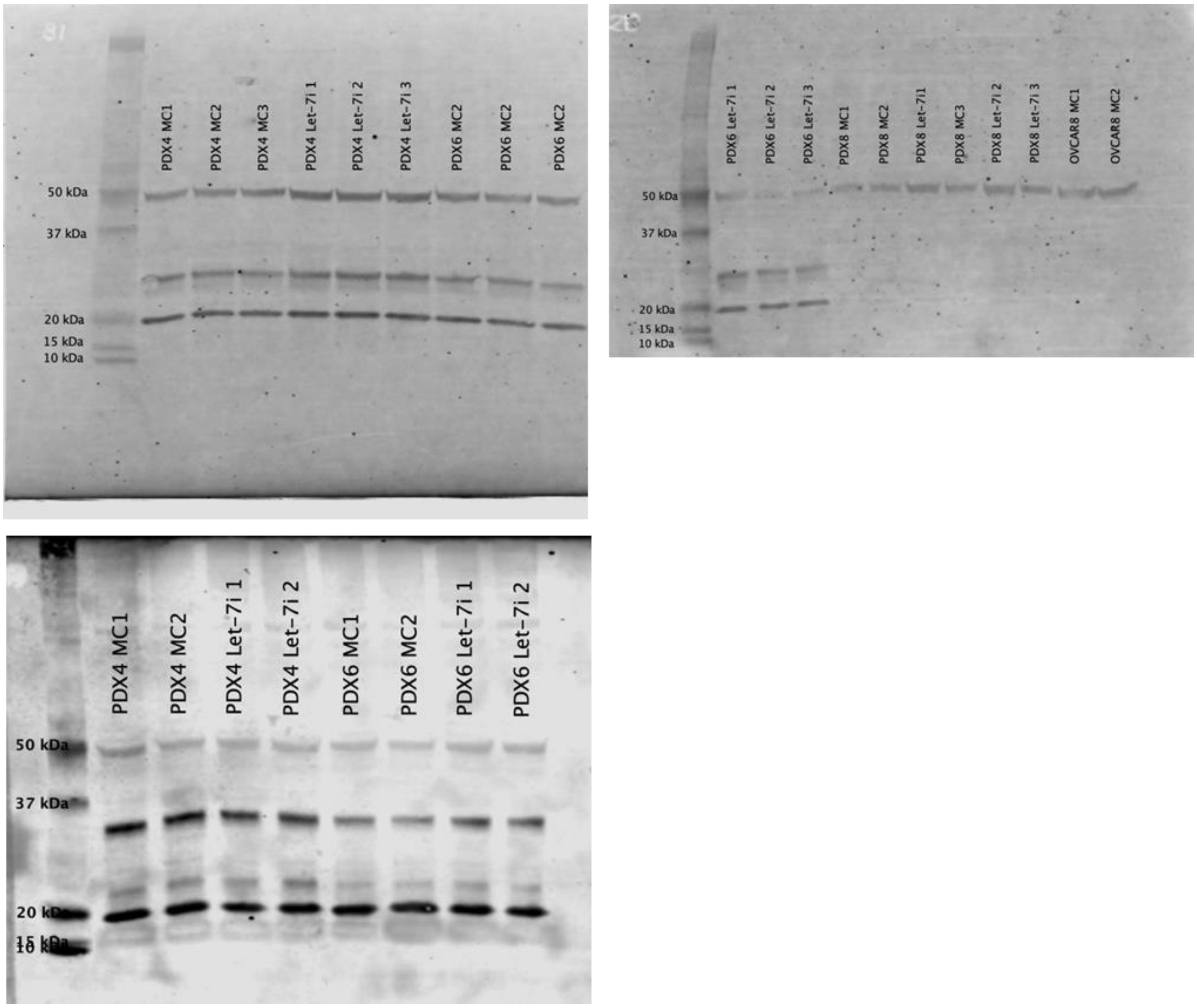

Figure S2. Full images of Western Blots staining for Tubulin, LIN28A, and HMGA2.

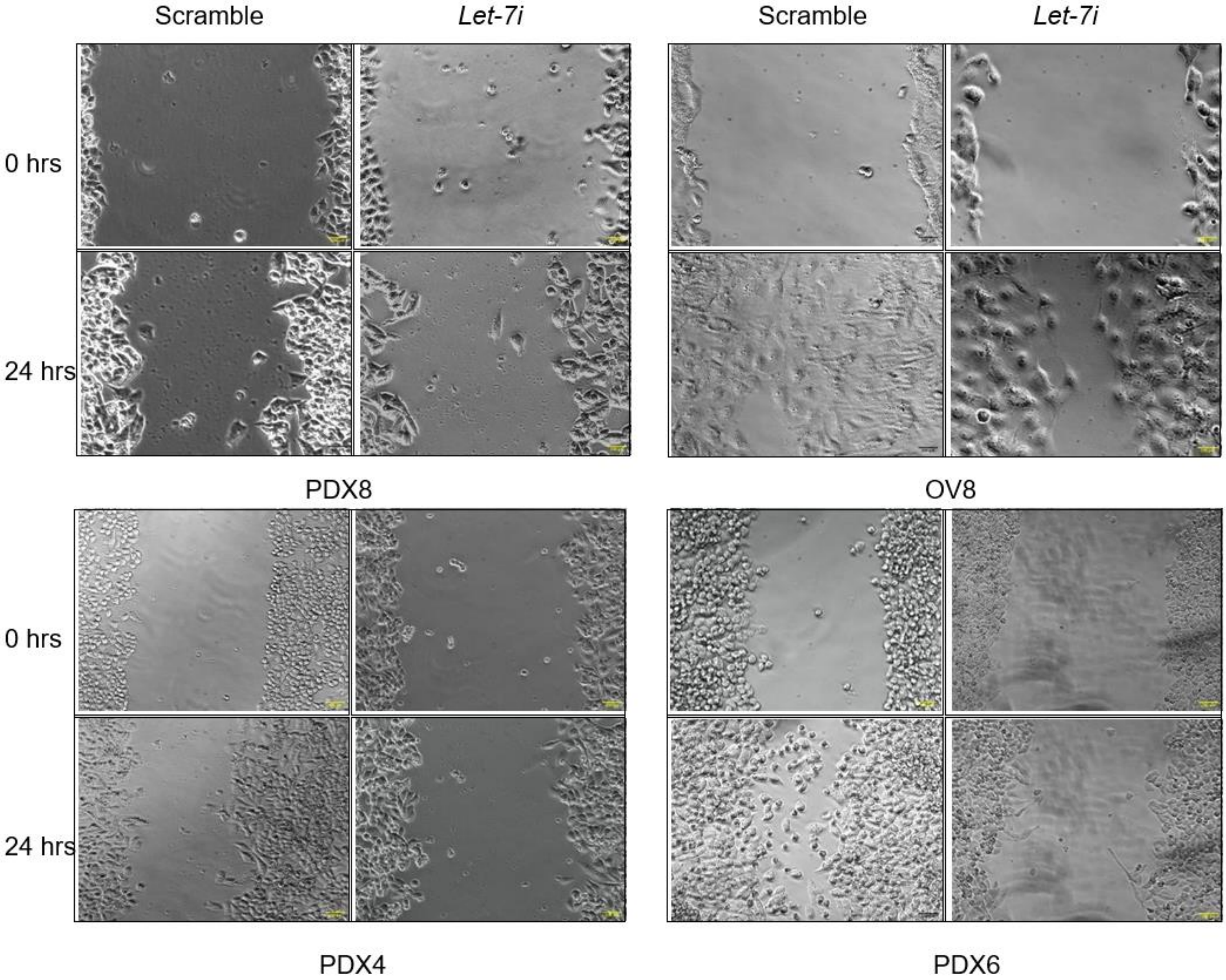

Figure S3. Images from wound healing assays. Representative images from control (Scramble) and let-7i overexpression for four cell types is shown.

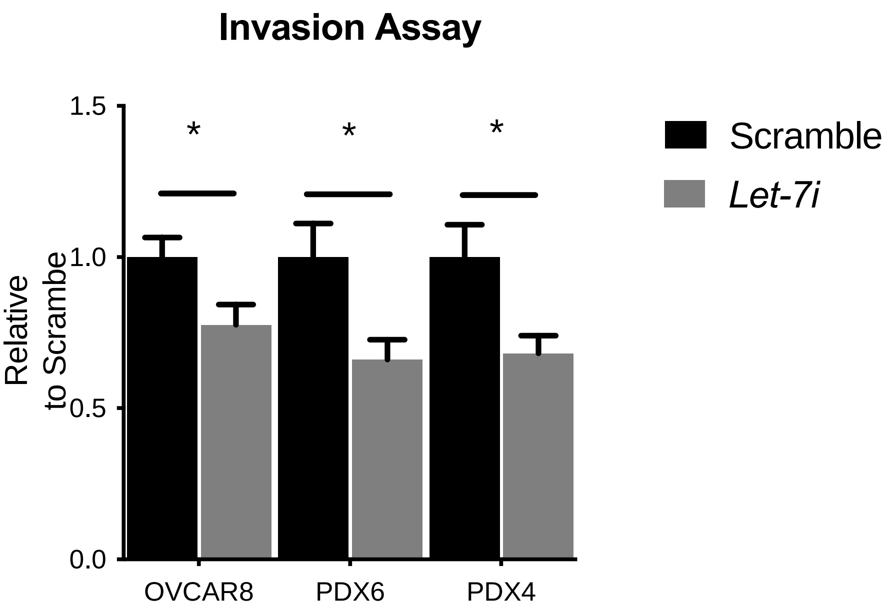

Figure S4. *Let-7* reduces invasion. Invasion assay performed 48 hours post *let-7i* over-expression. Error bars: SE. *$p \leq 0.05$. p *V*alue ≤ 0.05 was considered significant.

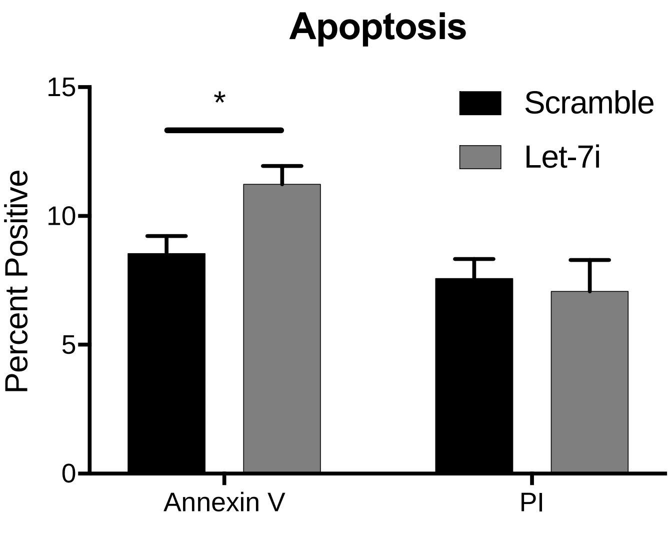

Figure S5. *Let-7i* increases apoptosis. Flow cytometry analysis was performed 48 hours post *let- 7i* over-expression in OVCAR8 cells and stained for Annexin V and PI to assess apoptosis. Error bars: SE. *$p \leq 0.05$. p Value ≤ 0.05 was considered significant.

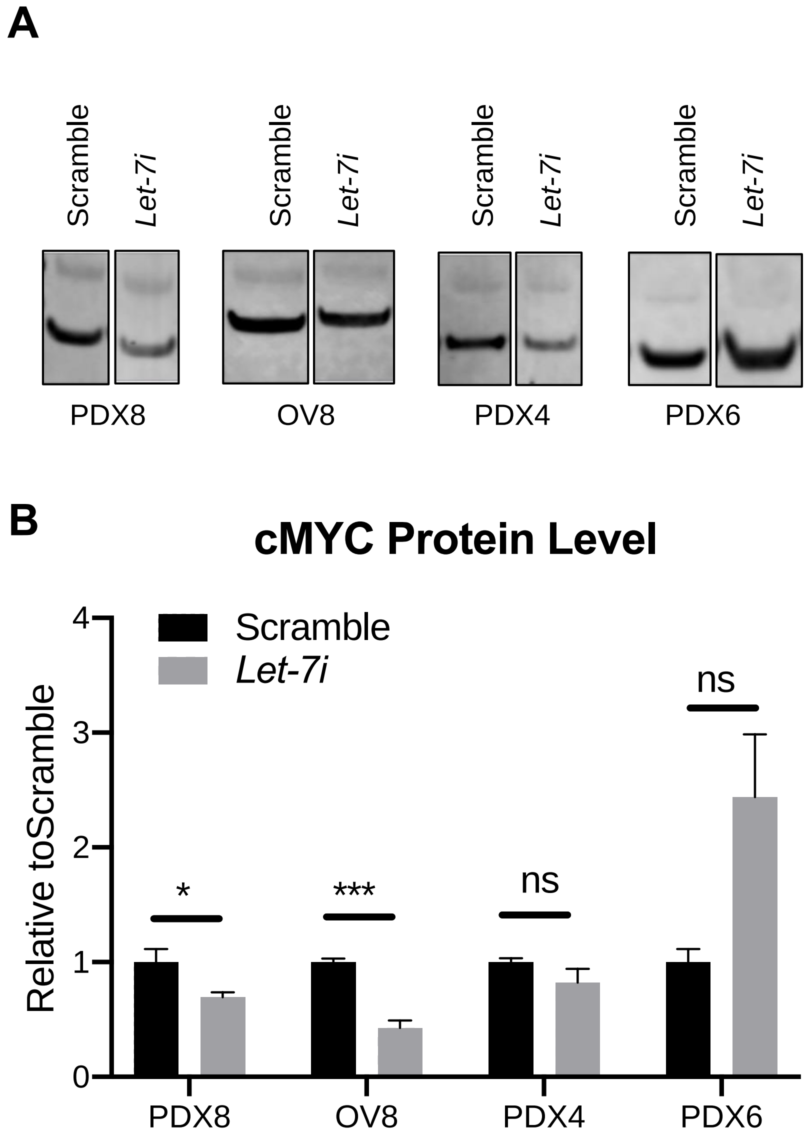

Figure S6. Let-7 effect on cMYC protein level. Let-7 OE resulted in cMYC repression in PDX8 and OV8 while having no effect on PDX4 and PDX6. *$p \leq 0.05$, ***$p \leq 0.001$. ns, not significant. p value ≤ 0.05 was considered significant.

Beta Actin F<br>Beta Actin R	TGAAGTGTGACGTGGACA TC<br>GGAGGAGCAATGATCTTGAT
kRAS F<br>kRAS R	CCCAGGTGCGGGAGAGA<br>TCAAGGCACTCTTGCCTACG
MYC F<br>MYC R	ACTCTGAGGAGGAACAAGAA<br>TGGAGACGTGGCACCTCTT
E2F1 F<br>E2F1 R	ACGCTATGAGACCTCACTGAA<br>TCCTGGGTCAACCCCTCAAG
LIN28A F<br>LIN28A R	GAGCATGCAGAAGCGCAGATCAAA<br>TATGGCTGATGCTCTGGCAGAAGT
IGF1 F<br>IGF1 R	GCTCTTCAGTTCGTGTGTGGA<br>CGACTGCTGGAGCCATACC
HMGA2 F<br>HMGA2 R	AAAACGGCCAAGAGGCAGAC<br>ATGTCTCTTCAGTCTCCTGAGCA

Table S1. RT-qPCR Primers. Sequences of forward and reward primers used for mRNA quantification.
